# Asociación entre la exposición a ruido ambiental y la calidad del sueño de adultos residentes en Medellín, Colombia, 2022: un estudio exploratorio

**DOI:** 10.1590/0102-311XES233423

**Published:** 2025-03-31

**Authors:** Oscar Alberto Rojas-Sánchez, Jonathan Ochoa-Villegas, Diana Marín, Juan Gabriel Piñeros-Jiménez, Laura Andrea Rodriguez-Villamizar

**Affiliations:** 1 Instituto Nacional de Salud, Bogotá, Colombia.; 2 Universidad de San Buenaventura, Medellín, Colombia.; 3 Universidad Pontificia Bolivariana, Medellín, Colombia.; 4 Universidad de Antioquia, Medellín, Colombia.; 5 Universidad Industrial de Santander, Bucaramanga, Colombia.

**Keywords:** Ruido, Efectos del Ruido, Salud Ambiental, Sueño, Calidad del Sueño, Noise, Noise Effects, Environmental Health, Sleep, Sleep Quality, Ruído, Efeitos do Ruído, Saúde Ambiental, Sono, Qualidade do Sono

## Abstract

El ruido ambiental es un contaminante urbano del aire en metrópolis. Nuestro objetivo fue determinar la asociación entre exposición diferencial a ruido ambiental y calidad del sueño de adultos. Se realizó estudio de corte transversal con muestreo aleatorio estratificado de viviendas según dos categorías de exposición (alta [≥ 65dBA] y baja [≤ 50dBA]), de acuerdo con mapa a priori de ruido de la ciudad. Se seleccionó personas con 18 o más años, que durmieran y residieran el mayor tiempo posible en la vivienda los últimos seis meses y, se excluyó a personas con tratamiento de alteraciones mentales o de la comunicación. Se utilizó un cuestionario auto diligenciado para medir las características sociodemográficas, clínicas, de estilo de vida, de exposición a ruido, y de calidad subjetiva del sueño (*Índice de Calidad de Sueño de Pittsburgh*). El análisis estadístico incluyó contrastes mediante chi cuadrado, correlaciones de Spearman y se estructuró un modelo de regresión de Poisson con varianza robusta. Los análisis se realizaron en Stata v12. Se incluyeron 221 participantes, 53,4% mujeres con edad mediana de 57 años (RIC: 35-67), 52,9% trabajadores y 45,9% con formación universitaria. El 47,5% de las personas tenía alguna patología de base. Los residentes en la zona de alta exposición presentaron 13% más prevalencia de mala calidad del sueño comparado con la zona de baja (RP = 1,13; IC95%: 0,99-1,28). El ruido ambiental altera la calidad del sueño, sin embargo, existen factores tales como la sensibilidad al ruido, la duración de la exposición, variables sociodemográficas, hábitos de vida y alteraciones en salud mental que modulan la manera y magnitud en que el ruido afecta dicha calidad del sueño.

## Abstract (en)

Environmental noise is an urban air pollutant in metropolises. This study aimed to identify the association between differential exposure to environmental noise and sleep quality in adults. This cross-sectional study used stratified random sampling of households according to two noise exposure categories (high [≥ 65dBA] and low [≤ 50dBA]), as specified by the urban noise map. People aged over 18 years, who slept and spent most of their time in the place during the last six months were selected, and people undergoing treatment for mental or communication disorders were excluded. A self-administered questionnaire was used to collect sociodemographic, clinical, lifestyle, noise exposure, and subjective sleep quality characteristics (*Pittsburgh Sleep Quality Index*). Statistical analysis included chi-squared tests and Spearman’s correlations, and a Poisson regression model with robust variance was used. The analyses were performed in Stata v12. A total of 221 participants were included, 53.4% female, with a mean age of 57 years (IQR: 35-67), 52.9% workers and 45.9% with higher education. It was observed that 47.5% of the participants had some underlying pathology. Residents of places with high noise exposure had a 13% higher prevalence of poor sleep quality compared to those in places with low exposure (PR = 1.13; 95%CI: 0.99-1.28). Although affecting sleep quality, the ways and extent with which environmental noise affects individuals seems to be influenced by noise sensitivity, length of exposure, sociodemographic variables, lifestyle habits, and mental health disorders.

## Resumo

O ruído ambiental é um poluente atmosférico urbano nas metrópoles. O objetivo deste estudo foi identificar a associação entre a exposição diferencial ao ruído ambiental e a qualidade do sono em adultos. Trata-se de um estudo transversal com amostragem aleatória estratificada de residências conforme duas categorias de exposição (alta [≥ 65dBA] e baixa [≤ 50dBA]), de acordo com o mapa do ruído urbano. Foram selecionadas pessoas maiores de 18 anos, que dormiam e moravam o maior tempo no local durante os últimos seis meses, e foram excluídas pessoas em tratamento de distúrbios mentais ou de comunicação. Foi utilizado um questionário autoaplicável para coletar as características sociodemográficas, clínicas, de estilo de vida, de exposição ao ruído e de qualidade subjetiva do sono (*Índice da Qualidade do Sono de Pittsburgh*). A análise estatística incluiu testes qui-quadrado e correlações de Spearman, e foi utilizado um modelo de regressão de Poisson com variância robusta. As análises foram realizadas no Stata v12. Foram incluídos 221 participantes, 53,4% do sexo feminino, com idade média de 57 anos (IIQ: 35-67), 52,9% trabalhadores e 45,9% com formação universitária. Foi observado que 47,5% dos participantes tinham alguma patologia subjacente. Os residentes de locais com alta exposição ao ruído tiveram uma maior prevalência de 13% de qualidade de sono ruim em comparação com aqueles de locais com baixa exposição (RP = 1,13; IC95%: 0,99-1,28). O ruído ambiental afeta a qualidade do sono, mas fatores como sensibilidade ao ruído, duração da exposição, variáveis sociodemográficas, hábitos de vida e distúrbios de saúde mental determinam a forma e a magnitude com que o ruído afeta a qualidade do sono.

## Introducción

La contaminación acústica ha emergido como uno de los principales problemas urbanos del siglo XXI, afectando tanto la calidad ambiental como la salud humana ^1^. La exposición continua al ruido, especialmente en entornos urbanos densamente poblados, ha sido vinculada a distintos problemas de salud, que van desde trastornos del sueño y estrés hasta enfermedades cardiovasculares ^2^ ^,^ ^3^. En particular, el sueño, un componente esencial para el bienestar y el rendimiento diario, se ha identificado como una de las áreas más afectadas por los altos niveles de ruido ambiental ^4^. Específicamente, se ha demostrado que el ruido fragmenta el sueño, reduce su continuidad y, como tal, el tiempo total de sueño efectivo ^5^ ^,^ ^6^.

La calidad del sueño es un factor de gran importancia para el mantenimiento de la salud de las personas y específicamente de la homeostasis, tanto corporal como cerebral ^7^ ^,^ ^8^. Esto implica un balance en el ciclo circadiano y una alineación de este con la homeostasis del sueño para prevenir problemas de tipo cardiovascular, metabólico, ansiedad, depresión u otros relacionados ^9^. En el año 2004, la Organización Mundial de la Salud (OMS) refería que una persona necesitaba dormir alrededor de 6 a 8 horas continuas para disminuir el riesgo de problemas de salud asociados ^10^ y una revisión de revisiones sistemáticas posterior confirma que se necesitan 7 a 8 horas para tener buena salud en la adultez ^11^. Además, dormir menos de 6 horas tiene la misma ponderación de riesgo que dormir más de 8 horas.

La relación entre el ruido ambiental y la calidad del sueño ha sido examinada en diversos contextos globales ^5^ ^,^ ^6^. Una revisión sistemática y meta-análisis publicada en 2022 por la OMS, evidenció que por cada 10dB de incremento en el ruido ambiental nocturno ocasionado por automotores, se aumentó en 1,52 veces (*odds ratio* [OR] = 2,52; intervalo de 95% de confianza [IC95%]: 2,28-2,79) la posibilidad de alteración del sueño de las personas. Los estudios incluidos en los análisis de la OMS fueron principalmente de países europeos, además de algunos norteamericanos y asiáticos ^6^.

En este contexto de abordaje de las relaciones entre exposición a ruido ambiental y salud existe escasez de investigaciones enfocadas a poblaciones latinoamericanas o del caribe, donde la rápida urbanización y las características únicas del paisaje sonoro pueden presentar escenarios diferenciales o particulares por sus condiciones de vida u otros determinantes en salud ^12^. Entre los pocos estudios disponibles en la región, la mayoría se han realizado en Brasil y Colombia. Estos han abordado efectos del ruido en instituciones hospitalarias ^13^, en grupos ocupacionales específicos ^14^ ^,^ ^15^ ^,^ ^16^ ^,^ ^17^, o en residentes alrededor de aeropuertos ^18^, pero no han abordado los efectos del ruido ambiental y sus efectos sobre la salud en el contexto de zonas urbanas.

Medellín, una ciudad colombiana con una diversidad de ambientes sonoros debido a su topografía y urbanización ^19^, proporciona un contexto ideal para investigar la relación entre ruido ambiental y sus efectos sobre la salud. El objetivo de este estudio fue determinar la asociación entre la exposición a ruido ambiental y la calidad del sueño en personas adultas residentes en un área urbana de la ciudad de Medellín durante los años 2021 y 2022. El propósito del estudio es proveer evidencia sobre las relaciones entre ruido ambiental y calidad de sueño en el contexto urbano de una ciudad latinoamericana. Nuestro estudio aporta nuevo conocimiento en esta área de la salud ambiental, área de investigación que tiene incipiente desarrollo en la región, y que es necesaria para informar la toma de decisiones de políticas locales y regionales, y el desarrollo de procesos de investigación en esta área de conocimiento. Cabe resaltar que actualmente la investigación, llevada a cabo en el Sur Global, es de relevancia en contraste con el Norte Global, ya que en este espacio geográfico se localizan gran parte de países con alto déficit de estudios científicos y con mayores deficiencias de normatividad regulatoria.

## Materiales y métodos

### Diseño epidemiológico, población, muestra y criterios de selección de la muestra

Se realizó un estudio observacional analítico de corte transversal con un muestreo probabilístico estratificado de los participantes. Se estimó un tamaño de muestra mínimo de 110 participantes en cada grupo de exposición para detectar diferencias en la prevalencia de mala calidad del sueño de al menos 20% entre los grupos de exposición, con un poder del 80% y error alfa de 5%. La unidad de muestreo fue la vivienda. Por lo tanto, las viviendas se estratificaron de acuerdo con los niveles de presión sonora continuos, equivalente para el periodo promedio de un año del *Mapa de Ruido Medellín*, año 2018 ^20^, y dentro de cada estrato se seleccionaron por muestreo aleatorio simple entre 100 a 120 viviendas, con la posibilidad de tener reemplazo si el potencial participante por vivienda no consintiera la participación en el estudio o no fuera elegible. En dichos casos, a partir del punto aleatorio elegido, se buscaba a la derecha o a la izquierda hasta lograr la inclusión efectiva de un participante.

Medellín es una ciudad colombiana, capital del departamento de Antioquia, con aproximadamente 2,6 millones de habitantes, siendo esta la ciudad más poblada del departamento y la segunda más poblada del país. Esta ciudad se encuentra a 1.479 metros de altitud, posee una extensión de 111,6km^2^ de suelo urbano y su temperatura promedio es de 24ºC. La división político-administrativa de la ciudad está conformada por 16 comunas ^21^.

**Figura 1:**
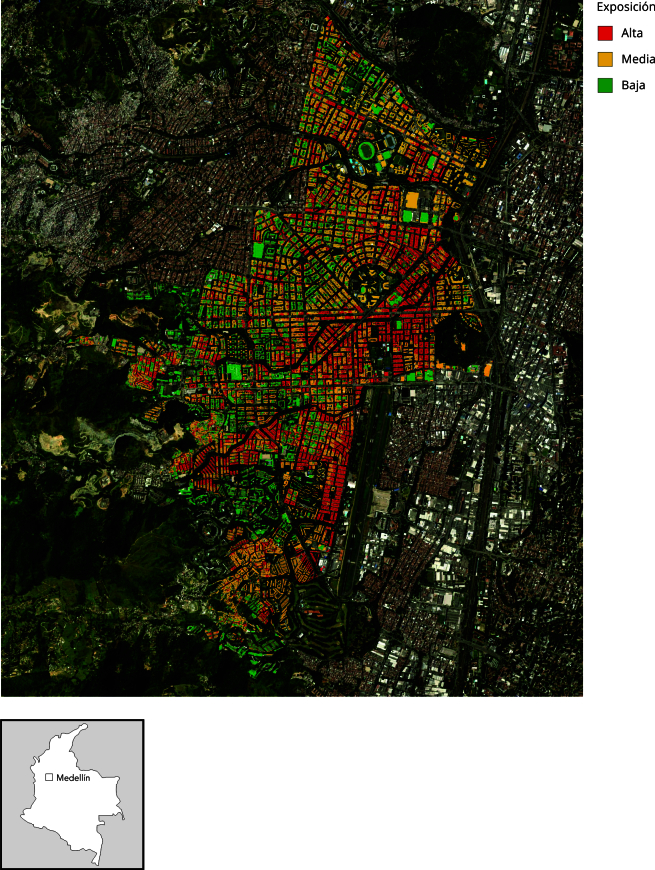
Georreferenciación de población y zonas de estudio. Comunas 11 y 16 de Medellín, Colombia.

Se seleccionaron como zonas de muestreo las comunas 11 y 16 (divididas por una avenida principal) de la ciudad de Medellín por incluir alta diversidad de niveles de exposición a ruido ambiental (Figura 1). Dentro de cada una de las comunas se tomó el mapa de predios del área de interés del estudio y se establecieron los dos estratos (baja exposición [≤ 50dBA] o alta exposición a ruido [≥ 65dBA]), según los niveles registrados por el mapa de ruido con detalle a nivel de predio (viviendas).

Nota: zonas de exposición georreferenciadas mediante ArcGIS v15 (http://www.esri.com/software/arcgis/index.html).

Los criterios de inclusión de las personas fueron: (i) ser mayor de 18 años; (ii) tiempo de residencia en el sector mayor a seis meses; (iii) ser residente habitual de la vivienda y que durmiera en la misma; (iv) permanencia el mayor tiempo en la vivienda; (v) residir en el área de interés del estudio (comunas 11 y 16 de Medellín), y en predios con alta o baja exposición a ruido ambiental, según mapa de ruido de la ciudad. Por su parte, los criterios de exclusión fueron: (i) presencia de alteración psicológica o psiquiátrica (auto-reporte de diagnóstico o tratamiento médico para depresión o ansiedad) y (ii) tener alteración en la comunicación que limitase su capacidad para responder preguntas o la evaluación del desenlace de interés.

### Medición del ruido ambiental

Para la medición de la exposición individual promedio, se tomó como referencia el mapa de ruido de la ciudad de Medellín que estimó niveles de presión sonora continuos equivalentes, para el periodo promedio del año 2018. Este mapa permitió caracterizar cada una de las viviendas de las dos zonas de exposición. En este caso se tomaron para el objeto del estudio los dos extremos, zonas con baja exposición (zonas verdes; ≤ 50dBA) y las de alta exposición a ruido (zonas rojas; ≥ 65dBA), buscando tener un margen de diferencia entre los dos puntos de comparación. De acuerdo con la normatividad colombiana (*Resolución 627 del 2006* ^22^), se han definido los estándares máximos permisibles de niveles de emisión de ruido para un Sector B (“zona de tranquilidad y ruido moderado”), que incluye zonas residenciales evaluadas en este estudio, los cuales oscilan entre 65dBA para el día y 55dBA para la noche.

### Medición de la calidad del sueño

Para establecer la calidad del sueño se utilizó el *Índice de Calidad de Sueño de Pittsburgh* (ICSP; *Pittsburgh Sleep Quality Index*), un cuestionario estandarizado con validación y utilización previa en Colombia ^23^. El índice posee siete componentes que evalúan la calidad subjetiva del sueño, su latencia, duración, eficiencia, perturbaciones, uso de sedación hipnótica y disfunción diurna en las actividades cotidianas de las personas. Además, posee 19 ítems auto-aplicados y cinco más diligenciados por el compañero(a) de habitación (en caso de tenerlo). Solo las preguntas auto-aplicadas están incluidas en el puntaje final. A cada componente se le asigna una puntuación entre 0 y 3 y, por tanto, los siete componentes se suman para obtener el puntaje total, que varía de 0 a 21 puntos. Una puntuación mayor a cinco representa un deterioro de la calidad del sueño. Por tanto, se establecieron dos categorías básicas de análisis: un puntaje menor de cinco fue asumido como “buena calidad del sueño o buenos dormidores” y un puntaje mayor o igual a cinco representó “pobre calidad del sueño o pobres dormidores” ^24^.

### Evaluación de potenciales confusores

El análisis de variables que pudieran desempeñar un efecto confusor sobre la asociación de interés, incluyó el desarrollo de un gráfico acíclico dirigido (DAG, por sus siglas en inglés) para establecer a priori las relaciones entre las variables explicativas captadas durante la recolección de los datos en campo. Dicho DAG también permitió el desarrollo del análisis estadístico (Material Suplementario - Figura S1; https://cadernos.ensp.fiocruz.br/static//arquivo/suppl-e00233423_8171.pdf). Mediante un cuestionario estandarizado (Material Suplementario; https://cadernos.ensp.fiocruz.br/static//arquivo/suppl-e00233423_8171.pdf) se obtuvo la información sociodemográfica y de estilo de vida, variables clínicas y percepción de exposición a ruido. En el cuestionario también se incluyó el test de Zung para síntomas de ansiedad, que incluye 20 ítems y una calificación total que puede variar entre 20 y 80 puntos. El cuestionario fue aplicado de manera presencial, con formato en papel y mediante diligenciamiento personal por cada participante (auto-aplicado) con acompañamiento durante su diligenciamiento para verificar dudas o completitud de la información.

### Análisis estadístico de los datos

Se realizó análisis estadístico de las características sociodemográficas, clínicas y de determinantes relevantes, que incluyó la utilización de medias o medianas y sus medidas de variación, frecuencias absolutas y relativas. En contraste bivariable se utilizaron las pruebas chi cuadrado y el coeficiente de Spearman para evaluar la correlación con mala calidad del sueño y en el análisis multivariable se realizó un modelo de regresión de Poisson con varianza robusta para el cálculo de la razón de prevalencias (RP). Se utilizó como variable desenlace la clasificación de calidad de sueño dicotomizada y el nivel de exposición a ruido como variable explicativa principal, ajustando por el efecto de otras variables potencialmente confusoras identificadas con el DAG. Se utilizó Stata, versiones 12.0 y 13.0 (https://www.stata.com), y se consideró significativo un valor de p bilateral de 0.05.

### Aspectos éticos de la investigación

El protocolo del estudio fue revisado y aprobado por dos comités de ética de la investigación de instituciones nacionalmente reconocidas: el Comité de Ética de la Universidad Industrial de Santander (CEINCI UIS; Acta 23 de 2019) y el Comité del Instituto Nacional de Salud (CEMIN INS; Acta 25 de 2019). Para la inclusión de los participantes al estudio se utilizó un consentimiento informado y se mantuvo durante la ejecución un estricto cumplimiento de los derechos de las personas y de los principios éticos.

## Resultados

En el estudio se incluyeron 221 participantes entre agosto de 2021 y octubre de 2022, de los cuales el 51,6% (n = 114) residían en viviendas ubicadas en zona de alta exposición y 76% (n = 168) correspondieron a residentes de la comuna 16. Para ello se visitaron 1.550 viviendas, tanto asignadas por el método de muestreo, como por los reemplazos ante casos de descarte por no cumplir criterios de selección, o no respuesta. El 11,4% (n = 177) se descartaron por tratarse de establecimientos comerciales, y el 74,1% (n = 1.148) de las viviendas visitadas o no se obtuvo respuesta (residencias vacías) o no se manifestó interés por participar por parte de los ocupantes. Las viviendas seleccionadas conservaron el nivel de exposición dentro del mismo bloque a las viviendas con no respuesta, en tanto que el reemplazo se realizó con la vivienda a derecha o izquierda de la vivienda inicial no efectiva.

La muestra estuvo conformada por 53,4% de mujeres, con mediana de edad de 57 años (rango intercuartílico [RIC]: 36-67), y un 53% de trabajadores activos. El 46,2% de las personas encuestadas tenía educación universitaria de pregrado o superior. Entre los antecedentes de importancia se encontró que el 31,3% fumaba o era ex fumador, el 69,7% consumía algún tipo de bebida estimulante como el café, el 23,1% había estado enfermo por COVID-19 y de ellos al menos el 33,3% había desarrollado síndrome post-COVID-19, el 58,8% realizaba al menos 30 minutos de ejercicio físico y principalmente moderado (70%), el 29,4% tenía algún tipo de patología de base como la hipertensión arterial (Tabla 1).

**Tabla 1:** Caracterización de la población de estudio según zona de exposición. Comunas 11 y 16 de Medellín, Colombia, 2021-2022.

Variables	Total [n (%)] N = 221	Zonas de exposición [n (%)]	
		Alta n = 114	Baja n = 107
Sexo			
Femenino	118 (53,4)	58 (50,9)	60 (56,1)
Masculino	103 (46,6)	56 (49,1)	47 (43,9)
Edad [mediana (RIC)]	57 (36-67)	52 (34-64)	60 (39-72)
Ocupación			
Estudiante	9 (4,1)	4 (3,5)	5 (4,7)
Ninguna	13 (5,9)	8 (7,0)	5 (4,7)
El hogar	32 (14,5)	11 (9,7)	21 (19,6)
Jubilado(a) o vivir de rentas	50 (22,6)	22 (19,3)	28 (26,2)
Trabajador independiente	57 (25,8)	33 (29,0)	24 (22,4)
Trabajador dependiente	60 (27,2)	36 (31,6)	24 (22,4)
Nivel educativo (más alto alcanzado)			
Primaria	21 (9,5)	7 (6,1)	14 (13,1)
Postgrado universitario	40 (18,1)	21 (18,4)	19 (17,8)
Secundaria	48 (21,7)	21 (18,4)	27 (25,2)
Técnica o tecnológica	50 (22,6)	29 (25,4)	21 (19,6)
Pregrado universitario	62 (28,1)	36 (31,6)	26 (24,3)
Estado civil			
Soltero(a)	90 (40,7)	53 (46,5)	37 (34,6)
Casado(a)	76 (34,4)	37 (32,5)	39 (36,5)
Unión libre	21 (9,5)	9 (7,9)	12 (11,2)
Divorciado(a) o separado(a)	18 (8,1)	10 (8,8)	8 (7,5)
Viudo(a)	16 (7,2)	5 (4,4)	11 (10,3)
Tipo de vivienda			
Apartamento	63 (28,5)	30 (26,3)	33 (30,8)
Casa	158 (71,5)	84 (73,7)	74 (69,2)
Estrato socioeconómico			
Medio-alto	92 (41,6)	53 (46,5)	39 (36,5)
Medio-bajo	83 (37,6)	38 (33,3)	45 (42,1)
Medio	44 (19,9)	23 (20,2)	21 (19,6)
Bajo	1 (0,5)	0	1 (0,9)
Alto	1 (0,5)	0	1 (0,9)
Horas exposición a pantallas [mediana (RIC)]	7 (4-12)	8 (5-14)	6,5 (3,5-11)
Consumo de cigarrillo			
Nunca	152 (68,8)	77 (67,5)	75 (70,1)
Ex-fumador	43 (19,5)	20 (17,5)	23 (21,5)
Actual	26 (11,8)	17 (14,9)	9 (8,4)
Consumo de café (té o bebidas energizantes; pocillos/día)			
Ninguno	67 (30,3)	34 (29,8)	33 (30,8)
1-3	115 (52,0)	64 (56,1)	51 (47,7)
4-6	32 (14,5)	12 (10,5)	20 (18,7)
7 o más	7 (3,2)	4 (3,5)	3 (2,8)
Ansiedad (test de Zung)			
Sí	52 (23,5)	26 (22,8)	26 (24,0)
Sueño (ICSP)			
Buenos dormidores (PC < 5)	43 (19,5)	20 (17,5)	23 (21,5)
Pobres dormidores (PC ≥ 5)	178 (80,5)	94 (82,5)	84 (78,5)
Actividad física			
≥ 30 minutos (sí)	130 (58,8)	66 (57,9)	64 (59,8)
Tipo de actividad			
Moderada	91 (70,0)	46 (69,7)	45 (70,3)
Intensa	8 (6,2)	5 (7,6)	3 (4,7)
Mixta	31 (23,9)	15 (22,7)	16 (25,0)
Enfermedades crónicas *			
Sí	65 (29,4)	29 (25,4)	36 (33,6)

ICSP: *Índice de Calidad de Sueño de Pittsburgh*; PC: punto de corte; RIC: rango intercuartílico.

* Enfermedades crónicas: diabetes, hipertensión, cáncer, hipotiroidismo, osteoporosis, enfermedad pulmonar obstructiva crónica, asma, artritis, entre otras.

Se encontró que el 80,5% de las personas fueron “pobres dormidoras” según el ICSP, de las cuales se tuvo una tendencia levemente superior en la zona de alta exposición (Tabla 1). De igual forma, esto fue concordante con la pregunta de autopercepción si el “ruido alteraba su sueño” (Tabla 2). Al realizar un análisis del desenlace de interés, calidad del sueño según el ICSP, no obstante, la relación entre zona y calidad del sueño no fue significativa (p = 0,46). Caso contrario sucedió con las variables ocupación (p = 0,06), estado civil (p < 0,01), tipo de vivienda (p = 0,03), síntomas de ansiedad, afectiva o somática, con test de Zung (p < 0,01), calidad de vida con test Eurohisqol-8 (p < 0,01); al igual que para los antecedentes de apnea del sueño (p = 0,06), antecedentes médicos de trastornos acústicos como tinnitus, hipoacusia o sordera (p = 0,05) y consulta médica en el mes previo a la inclusión en el estudio (p = 0,04) (Tabla 3). De igual modo, se encontró correlación entre la edad con el nivel de ruido (p = 0,02) y además con la calidad del sueño (p = 0,02). También se observó una correlación de gran magnitud entre edad y tiempo de uso de pantallas (p < 0,01).

**Tabla 2:** Características de la exposición a ruido y de la calidad del sueño. Comunas 11 y 16 de Medellín, Colombia, 2021-2022.

Variables	Total [n (%)] N = 221	Zonas de exposición [n (%)]	
		Alta n = 114	Baja n = 107
Ruido externo más molesto (los dos más frecuentes)			
Tráfico vehicular (o rodado)	77 (34,8)	39 (34,2)	38 (35,5)
Actividades de ocio (bares, discotecas o fiestas clandestinas)	53 (24,0)	30 (26,3)	23 (21,5)
Grado de molestia percibido al ruido ambiental externo			
Alta o muy alta	157 (71,0)	86 (75,4)	71 (66,4)
Baja o ninguna	64 (29,0)	28 (24,6)	36 (33,6)
Existencia de algún ruido interno molesto			
Sí	113 (51,1)	56 (49,1)	57 (53,2)
El ruido al que está expuesta la persona es principalmente externo			
Siempre	104 (47,1)	55 (48,3)	49 (45,8)
Casi siempre	66 (29,9)	40 (35,1)	26 (24,3)
Algunas veces	37 (16,7)	15 (13,2)	22 (20,6)
Casi nunca	8 (3,6)	3 (2,6)	5 (4,7)
Nunca	6 (2,7)	1 (0,9)	5 (4,7)
Nivel de exposición a ruido ambiental (dBA) [X̅ (DE)]	57,5 (9,3)	66,3 (1,2)	48,1 (2,0)
Alteración del sueño por el ruido			
Sí	105 (47,5)	61 (53,5)	44 (41,1)
Habitación de dormir			
Exposición a luz (al dormir)			
Alta o muy alta	64 (29,0)	37 (32,5)	27 (25,2)
Baja o ninguna	157 (71,0)	77 (67,5)	80 (74,8)
Zona verde (frente a habitación de dormir)			
No	190 (86,0)	100 (87,7)	90 (84,1)
Vía de alto flujo (cerca a habitación de dormir)			
No	149 (67,4)	66 (57,9)	83 (77,6)

dBA: decibeles ponderados; DE: desviación estándar; X̅: promedio o media.

**Tabla 3:** Análisis del evento de interés, calidad del sueño según el *Índice de Calidad de Sueño de Pittsburgh* (ICSP). Comunas 11 y 16 de Medellín, Colombia, 2021-2022.

Variables	ICSP		Valor de p *
	Buenos dormidores n = 43	Pobres dormidores n = 178	
Sexo			0,17
Femenino	27	91	
Masculino	16	87	
Ocupación			0,06
Estudiante	4	5	
Ninguna	0	13	
El hogar	3	29	
Jubilado(a) o vive de rentas	9	41	
Trabajador independiente	12	48	
Trabajador dependiente	15	42	
Nivel educativo (más alto alcanzado)			0,45
Primaria	4	17	
Postgrado universitario	11	29	
Secundaria	7	41	
Técnica o tecnológica	7	43	
Pregrado universitario	14	48	
Estado civil			< 0,01
Soltero(a)	30	60	
Casado(a)	9	67	
Unión libre	3	18	
Divorciado(a) o separado(a)	0	18	
Viudo(a)	1	15	
Tipo de vivienda			0,03
Apartamento	18	45	
Casa	25	133	
Estrato socioeconómico			
5	24	68	0,31
3	13	70	
4	6	38	
2	0	1	
6	0	1	
Exposición a ruido			
Ruido externo más molesto			0,72
Tráfico vehicular (o rodado)	14	63	
Actividades de ocio (bares, discotecas o fiestas clandestinas)	11	42	
Actividades de construcción	6	12	
Industrias	2	9	
Comercio	2	6	
Tráfico aéreo	4	17	
Otros	4	29	
Grado de molestia percibido al ruido ambiental externo			0,04
Alta o muy alta	25	132	
Baja o ninguna	18	46	
Alteración del sueño por el ruido			0,24
Sí	17	88	
No	26	90	
Habitación de dormir			
Exposición a luz (al dormir)			0,84
Alta o muy alta	13	51	
Baja o ninguna	30	127	
Zona verde (frente a habitación de dormir)			0,99
Sí	6	25	
No	37	153	
Vía de alto flujo (cerca a habitación de dormir)			0,47
Sí	16	56	
No	27	122	
Consumo de cigarrillo			0,14
Nunca	35	117	
Ex-fumador	5	38	
Actual	3	23	
Consumo de café (té o bebidas energizantes, pocillos/día)			0,24
Ninguno	17	50	
1-3	22	93	
4-6	4	28	
7 o más	0	7	
Ansiedad (test de Zung)			< 0,01
Sí	0	52	
No	43	126	
Depresión (*proxy* de test de Zung)			0,20
Sí	1	14	
No	42	164	
COVID-19			0,66
Sí	11	40	
No	32	138	
Síndrome post-COVID-19			0,63
Sí	3	14	
No	8	26	
IMC (ajustado según el NHANES III)			0,54
Bajo (déficit)	7	42	
Medio (adecuado)	24	86	
Alto (exceso)	12	50	
Actividad física			0,43
Sí	23	107	
No	20	71	
Tipo de actividad			0,84
Moderada	16	75	
Intensa	2	6	
Mixta	5	26	
Calidad de vida (puntaje EUROHIQoL-8 con PC = 32)			< 0,01
Regular o buena	11	93	
Muy buena	32	85	
Percepción de afectación de la calidad de vida por ruido			0,17
Afectación alta	17	91	
Afectación baja	26	87	
Patologías de base			0,17
Sí	9	56	
No	34	122	
Apnea del sueño			0,06
Sí	0	14	
No	43	164	
Problemas de los oídos			0,05
Sí	1	22	
No	42	178	
Cita médica (último mes)			0,04
>Sí	13	85	
>No	30	93	
Zona de exposición			0,46
Alta (≥ 65dBA)	20	94	
Baja (≤ 50dBA)	23	84	

dBA: decibeles ponderados; EUROHIQoL-8: Pesquisa de Entrevistas de Saúde Europeu - Instrumento Abreviado de Qualidade de Vida; IMC: índice de masa corporal; PC: punto de corte.

* Test chi cuadrado.

La autopercepción sobre el “grado de molestia al ruido externo a la casa” estuvo correlacionada con ser pobre dormidor (p = 0,04) (Tabla 3). Entre los dos principales ruidos externos más molestos se encontraron en primer lugar el tráfico vehicular (o rodado) con 34,8% de los auto-reportes, seguido de las actividades de ocio (bares, discotecas o fiestas clandestinas) con 24%. El grupo de personas con mayor exposición presentó un grado de molestia percibido al ruido ambiental externo alto o muy alto, 9% mayor comparado con las personas de baja exposición; además, este mismo grupo manifestó 12,4% mayor alteración del sueño por el ruido en comparación con el grupo de baja exposición (Tabla 2).

**Figura 2:**
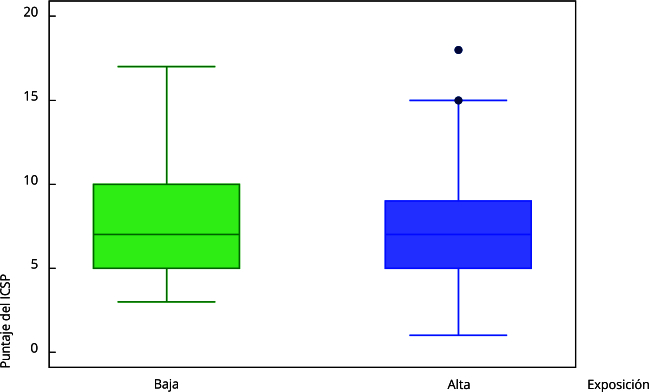
Relación gráfica entre los niveles de exposición a ruido ambiental promedio y el puntaje del *Índice de Calidad de Sueño de Pittsburgh* (ICSP). Medellín, Colombia, 2021-2022.

En cuanto a los niveles de ruido ambiental (LAeq,T: nivel de presión sonora continuo equivalente para el tiempo de medición T) se encontró un promedio de 66,3dBA (desviación estándar [DE]: 1,2) en la zona de alta exposición y 48,1dBA (DE: 2,0) en la de baja exposición. Al graficar la distribución del ICSP según las zonas de exposición, se encontró una distribución muy homogénea entre la varianza de la calidad del sueño para las dos zonas de exposición (Figura 2).

En el análisis multivariable, se evidenció diferencias entre las zonas de exposición y su efecto sobre la calidad del sueño, con una significación estadística marginal (RP = 1,13; IC95%: 0,99-1,28). Los resultados finales indican que los residentes en la zona de alta exposición a ruido ambiental (≥ 65dBA) tienen una prevalencia 13% más alta de mala calidad del sueño comparado con la zona de baja exposición. Este modelo se ajustó por sexo, edad (> 60 años), ocupación, estado civil, horas de exposición a pantallas, cercanía al tráfico de la habitación para dormir, realización de actividad física y puntaje de Zung para ansiedad (puntaje > 36) (Tabla 4).

**Tabla 4:** Modelo multivariable de la asociación entre exposición crónica a ruido ambiental y calidad del sueño. Comunas 11 y 16 de Medellín, Colombia, 2021-2022.

**Calidad del sueño ajustada [pobres dormidores (ICSP: PC ≥ 5) *vs.* buenos dormidores]**	RP	IC95%	Valor de p
Zona de exposición [Alta]	1,13	0,99-1,28	0,08
Sexo [Masculino]	1,23	1,07-1,43	< 0,01
Edad [Mayor de 60 años]	0,95	0,82-1,10	0,52
Ocupación 1 [Estudiante; trabajador dependiente o trabajador independiente]	0,77	0,66-0,91	< 0,01
Ocupación 2 [Jubilado(a) o vive de rentas]	0,89	0,76-1,04	0,15
Estado civil 2 [Divorciado(a) o separado(a)]	1,06	0,92-123	0,40
Estado civil 3 [Soltero(a)]	0,79	0,67-0,93	< 0,01
Estado civil 4 [Unión libre]	1,05	0,86-1,28	0.63
Estado civil 5 [Viudo(a)]	1,17	0,98-1,38	0,08
Tiempo de exposición a pantallas (celular u otros) [> 15 horas]	1,14	0,95-1,37	0,17
Cercanía de la habitación para dormir a vía con alto flujo vehicular	0,89	0,77-1,03	0,12
Actividad física [Sí]	1,10	0,95-1,26	0,20
Ansiedad [test de Zung > 36]	1,32	1,20-1,47	< 0,01

IC95%: intervalo de 95% de confianza; ICSP: *Índice de Calidad de Sueño de Pittsburgh*; PC: punto de corte; RP: razón de prevalencias.

## Discusión

Los resultados de nuestro estudio sugieren que existe una mayor alteración de la calidad del sueño en las personas residentes en dos comunas de la ciudad de Medellín que están expuestas a niveles crónicos de ruido ambiental, iguales o superiores a 65dBA. Nuestros hallazgos aportan información relevante para comprender la relación entre la exposición al ruido y la calidad del sueño en entornos urbanos de Colombia. Hasta nuestro conocimiento, este es el primer estudio latinoamericano que aborda esta relación usando una aproximación analítica que controla posibles efectos de confusión.

En el contexto global, los altos niveles de contaminación sonora en entornos urbanos han sido consistentemente asociados con perturbaciones en la calidad del sueño ^5^ ^,^ ^25^, pero esta relación ha sido evidenciada principalmente en países de Europa y es menos explorada en países de medianos y bajos ingresos como los de Latinoamérica. En el contexto nacional, en Bogotá se ha avanzado desde 2013 en un sistema de vigilancia de salud ambiental que incluye alteraciones en salud por exposición a ruido y que incluye alteraciones en la calidad del sueño. Como producto de la vigilancia, se informó que para 2021 el 23% de los participantes en la encuesta anual reportaron alteraciones con tres o más síntomas en relación con su exposición a ruido (SaluData Bogotá ^26^). Si bien esta información es útil para la caracterización de esta problemática a nivel local, su análisis es eminentemente descriptivo y no aborda sus posibles asociaciones. Nuestro estudio aporta nuevo conocimiento en el avance de la caracterización de la asociación entre exposición a ruido ambiental y la prevalencia de alteraciones del sueño.

Nuestros resultados muestran que el 80,5% de los participantes se clasificaron como “pobres dormidores” de acuerdo con el puntaje de la ICSP, mostrando una alta prevalencia de las alteraciones del sueño en el entorno urbano. Estos hallazgos son similares a los reportados en otros estudios de la región y el país, entre los que se destacan el realizado por Zamorano González et al. ^27^, en las zonas de mayor afluencia vial de Matamoros (Tamaulipas, México) quienes reportaron problemas para dormir en 55,3% de una muestra de 732 personas utilizando la misma escala de medición de calidad del sueño. Del mismo modo, el estudio de Callejas et al. ^18^, realizado en la zona de influencia del aeropuerto El Dorado de Bogotá, en el que se reportó que el 60% de los participantes tuvieron una mala calidad del sueño. De manera similar, un estudio en estudiantes universitarios reportó una prevalencia de “pobres dormidores” del 58% ^28^. Por su parte, el sistema de vigilancia ambiental de Bogotá reportó que para 2021 la prevalencia de alteraciones del sueño fue de 40% (SaluData Bogotá). Las prevalencias reportadas por estudios previos son inferiores a la reportada en nuestro estudio y puede estar relacionada con diferencias en el muestreo y las características de las poblaciones estudiadas, pero puede ser también un indicio del deterioro de la calidad del sueño progresiva.

La percepción subjetiva de la molestia del ruido estuvo muy correlacionada con el nivel de exposición a ruido ambiental. Se ha descrito previamente que la percepción de la molestia podría actuar como un mediador entre el ruido y la perturbación del sueño. Las principales fuentes de ruido ambiental relacionadas con molestias fueron el tráfico vehicular y las actividades de ocio, que se presentan como fuentes de interés para las intervenciones de políticas públicas. Estos hallazgos son consistentes con los de estudios realizados en provincias de Chile y más recientemente en la ciudad de São Paulo (Brasil), en los que se reportó el tránsito como una de las fuentes principales de molestia por ruido ^29^ ^,^ ^30^. Particularmente, en São Paulo se documentó una relación dosis-respuesta entre el nivel de ruido por tráfico y el nivel de molestia percibida ^30^. En Bogotá, el sistema de vigilancia de salud ambiental también ha documentado que, en esa ciudad, el ruido de tráfico vehicular es la fuente que produce mayor molestia (46%) seguido del pregoneo (34%) (SaluData Bogotá). A diferencia de lo reportado en estas ciudades, nuestros resultados en las zonas de estudio en Medellín muestran que después del ruido relacionado con el tráfico, el ruido producido por las actividades de ocio es el segundo más molesto, siendo un aspecto distintivo que puede estar relacionado con las características de la zona de estudio y no necesariamente puede generalizarse a toda la ciudad y todos los entornos urbanos. No obstante, Medellín, al igual que otras ciudades como Bogotá, Cali o Cartagena, son ciudades con alto turismo y esto trae consigo un aumento de la oferta de actividades nocturnas.

Es importante destacar la correlación encontrada entre la edad, el nivel de ruido y la calidad del sueño. A medida que las personas envejecen, pueden volverse más sensibles al ruido o, alternativamente, otros factores asociados con la edad tales como el consumo de café o cigarrillo, el índice de masa corporal, nicturia o enfermedades crónicas, podrían exacerbar las interrupciones del sueño. Las correlaciones encontradas con el tiempo de uso de pantallas y la ansiedad también resaltan la complejidad de la relación entre el ruido y la calidad del sueño. En particular, la fuerte correlación entre el uso de pantallas y la edad sugiere que las generaciones más jóvenes podrían estar más expuestas a perturbaciones del sueño debido a sus hábitos de uso de la tecnología. No obstante, aunque nuestro estudio evaluó algunos factores protectores, que menguan el influjo de tales factores, como vivir cerca o alrededor de zonas verdes que producen “un efecto conciliador o antagonista” y que alejan a las personas de las fuentes de ruido, esto no fue significativo estadísticamente. Contrario a lo encontrado por nuestra investigación, en una investigación muy reciente realizada en adolescentes ^31^, se encontró que vivir cerca de espacios verdes disminuye las probabilidades de no dormir al menos 8 horas (OR = 0,86; IC95%: 0,71-1,05).

Entre las principales limitaciones de nuestro estudio se encuentran su diseño transversal, el tamaño de muestra y la falta de información de estimaciones de ruido ambiental en las franjas horarias nocturnas para su análisis. El diseño transversal limita conocer las variaciones temporales de la calidad del sueño y su relación con variaciones temporales de corto y largo plazo del ruido ambiental. Adicionalmente, la naturaleza transversal limita asumir asociaciones causales, dada que la temporalidad de la relación no es clara. Nuestro estudio fue diseñado como un estudio piloto para identificar diferencias en las alteraciones del sueño iguales o superiores a 20% entre los grupos de exposición a ruido. Los hallazgos de prevalencias en los grupos de exposición y de intervalos de confianza marginales en los estimados de efecto, evidencian que las diferencias intraurbanas son menores y estudios futuros con tamaños de muestra mayores y diseños prospectivos pueden dar estimaciones más precisas de la relación entre ruido ambiental y calidad de sueño.

Por su parte, como una de nuestras limitaciones, utilizamos las estimaciones promedio diarias del mapa de ruido ambiental de la ciudad, que en su modelamiento incluyó mediciones diurnas y nocturnas, pero no desagregadas, por lo cual no fue posible evaluar la asociación de los niveles nocturnos de ruido exclusivamente con la alteración del sueño de los participantes, relación que de acuerdo con la literatura pudiera tener una mayor fuerza de asociación que las encontradas en nuestros resultados. Es importante resaltar aún con estas limitaciones que nuestros resultados sugieren una asociación positiva entre exposición a niveles altos de ruido y alteraciones del sueño en adultos. Si bien no se puede inferir una relación causal directa a partir de estos resultados, ofrecen una base para investigaciones más detalladas y experimentos controlados en el futuro.

Entre las principales fortalezas de este estudio se destaca la calidad del proceso de recolección de información y su abordaje analítico con control de posibles sesgos y confusión, El estudio incluyó el uso de un cuestionario estandarizado y validado para evaluar la calidad de sueño ^23^ y la medición objetiva de ruido ambiental mediante los registros detallados a nivel de predios del mapa de ruido disponible en Medellín. Adicionalmente, el abordaje analítico orientado por diagramas acíclicos directos permitió identificar posibles fuentes de sesgos de selección y efectos de confusión que fueron controlados en el diseño y el análisis multivariable.

Una consideración especial relacionada con nuestra elección del instrumento utilizado ^24^ para medir la autopercepción de calidad del sueño es que, aunque eventualmente pudiera existir una discrepancia potencial con estudios que utilicen herramientas más objetivas para medir el sueño o trastornos relacionados, tales como actigrafía y polisomnografía, habría, sin embargo, una ventaja del uso del ICSP es que se evalúan de manera integral siete componentes, da una mirada suficientemente amplia de la calidad del sueño, y que además puede utilizarse tanto en ambientes clínicos como no clínicos. Por tanto, aunque los instrumentos de medición objetiva pudieran dar una medida más precisa de la afectación biológica del sueño, el ICSP es una herramienta de evaluación subjetiva de calidad del sueño con una amplia validación internacional que provee información valiosa en estudios epidemiológicos.

Los hallazgos de nuestro estudio recalcan la necesidad de considerar una variedad de factores de relevancia cuando se estudia la calidad del sueño y su relación con el entorno sonoro. Factores como la edad (mayores de 60 años), ocupación (estudiante, trabadores dependientes o independientes), estado civil (soltero o viudo), tiempo de exposición a pantallas (> 15 horas), cercanía de la habitación para dormir a vía con alto flujo vehicular y ansiedad fueron identificados como relacionados con la calidad del sueño en este estudio y desempeñan un papel preponderante en cómo los individuos experimentan y se adaptan al ruido ambiental.

En conclusión, la exposición crónica a altos niveles de ruido ambiental se documentó como una condición asociada con pobre calidad del sueño en las zonas de estudio de Medellín. Adicionalmente, se evidencia que, la percepción individual del ruido y otros factores individuales y ecológicos pueden ser igualmente cruciales. Las intervenciones futuras en Medellín y en otras ciudades similares deberían considerar estos hallazgos al formular políticas y estrategias para mejorar la salud pública relacionada con el sueño.
