# Supplementary material for: Association between environmental noise exposure and sleep quality in adults living in Medellín, Colombia, 2022: an exploratory study
Source: Cad Saude Publica. 2025 Mar 31;41(2):e00233423. [Article in Spanish] doi: 10.1590/0102-311XES233423 (PMC11960757; doi:10.1590/0102-311XES233423)
Supplement: Supplementary file 1 [file 1678-4464-csp-41-02-ES233423-s.pdf]

MATERIAL SUPLEMENTARIO

Figura S1 Diagrama acíclico dirigido de la relación entre exposición a ruido ambiental y calidad de sueño.

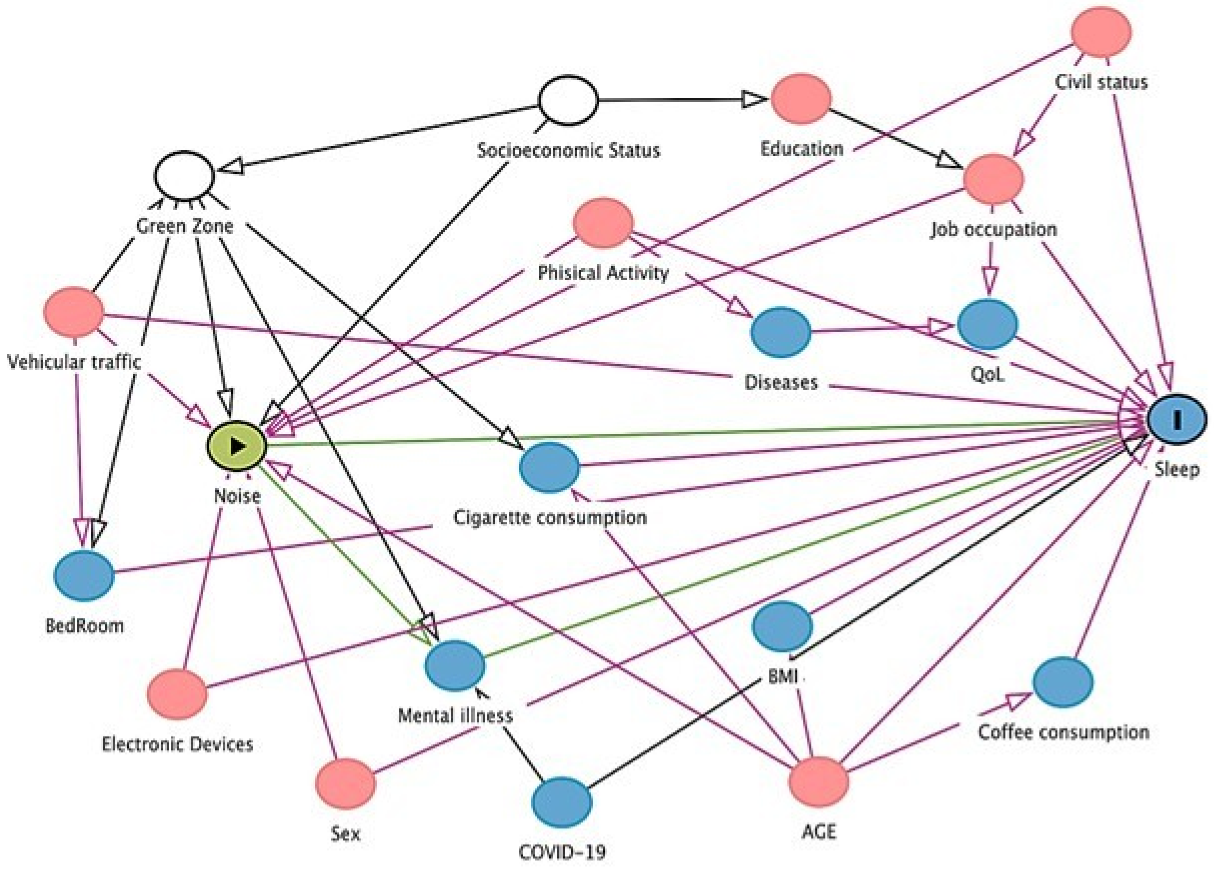

El conocimiento es de todos · Minciencias	**CALIDAD DEL AIRE Y SALUD AMBIENTAL URBANA EN CINCO CIUDADES DE COLOMBIA**<br>Código MINCIENCIAS 110284268242	
	**Cuestionario compilado Ruido y Salud**	Código: FO-HIRUIDO<br>Versión: 03<br>Fecha: 07/2021

CÓDIGO DE LA PERSONA

ID			zona	

**IDENTIFICACIÓN**	
**1. Nombre completo:**	
**2. Número de cédula:**	
**3. Fecha y hora de entrevista:**	
**4. Dirección de residencia:**	
**5. Tipo de vivienda**	**6. Estrato de la vivienda**
Casa: ______ Apartamento: ______	1___ 2___ 3___ 4___ 5___ 6____
**7. Teléfono Fijo:**	**8. Celular**
**9. Edad (años cumplidos)**	**10. Fecha de nacimiento:** DD/MM/AAAA

Diligenció: ____________________________

<table>
<tr><td rowspan="2"></td><td colspan="2">CALIDAD DEL AIRE Y SALUD AMBIENTAL URBANA EN CINCO CIUDADES DE COLOMBIA
Código MINCIENCIAS 110284268242</td></tr>
<tr><td>Cuestionario compilado Ruido y Salud</td><td>Código: FO-HIRUIDO
Versión: 03
Fecha: 07/2021</td></tr>
</table>

CÓDIGO DE LA PERSONA

ID			zona	

CARACTERÍSTICAS SOCIODEMOGRÁFICAS

1. Sexo:
Femenino___ Masculino___ Otro ___

2. Ocupación:
Sin trabajo___
Trabajador dependiente___
Trabajador independiente___
Jubilado(a) o vive de rentas___
Estudiante___
El hogar___

3. Nivel educativo (más alto alcanzado):
Ninguno___ Primaria___
Secundaria___ Técnica o tecnológica___
Pregrado universitario___
Postgrado universitario __

4. Estado civil:
Unión libre___ Casado(a)___
Divorciado(a) o separado(a)___
Viudo(a)___ Soltero(a)___

CARACTERISTICAS CLINICAS Y ESTILOS DE VIDA

5. Indique si ha sido diagnosticado alguna vez en la vida por un médico o presenta alguna de las siguientes condiciones de salud:
EPOC Sí ___ No___
Asma Sí ___ No___
Falla cardiaca Sí ___ No___
Artritis Sí ___ No___
Depresión Sí ___ No___
Ansiedad Sí ___ No___
Apnea del sueño Sí ___ No___
Despertar con ahogo Sí ___ No___
Piernas inquietas Sí ___ No___
Problema de oídos Sí ___ No___
Otra de importancia (cuál? si son varias separar concomas):________________________________
____

6. ¿Toma algún de medicamento de forma frecuente?
Si___ No___
Cuál(es): ___________________________________

7. **¿Realiza alguna actividad física** (mayor de 30 minutos al día)?
Sí ___ No___

8. En una semana típica, ¿Cuántos días realiza usted actividad física?
Total de días: ____

9. Tipo de actividad física realizada:
Actividad física moderada: Sí ___ No___
Actividad física intensa: Sí ___ No___
Actividad física MIXTA: Sí ___ No___

Nota: Actividad Intensa implica un aumento importante de la respiración o del ritmo cardíaco (gran esfuerzo físico como correr, pedalear, o nadar rápido, entre otras) y en la moderada es leve o poco (montar en bicicleta, trotar a paso regular, nadar despacio, bailar a ritmo suave, entre otras).

10. **Tiempo de actividad física o recreativa moderada durante la última semana** (sumar las actividades mayores a 10 minutos) ________ minutos/semana

11. **Tiempo de actividad física o recreativa vigorosa durante la última semana** (sumar las actividades mayores a 10 minutos) ________ minutos/semana

12. **Horas de exposición a pantallas en un día habitual** (si es más o menos de la hora colocarlo en decimales y si no aplica cero)**:**

    Televisión Horas: ___
    Computador Horas: ___
    Celular Horas: ___
    Tablet Horas: ___

13. **Número de pocillos de café, té o bebidas energizantes que consume al día** (si no toma colocar cero): ____

14. **Fumador**
Nunca___ Exfumador ___ Actual ____

15. **Número de cigarrillos promedio al día:** ______

16. **¿Ha tenido cita médica durante el último mes?** Sí___ No___

Motivos de consulta:
____
____

17. **Peso**: ____ (Kg)
18. **Talla**: ____(cms)

19. **¿Usted ha enfermado por Coronavirus o Covid-19?** Sí___ No___

20. **¿Hace cuantos meses tuvo Coronavirus o Covid-19? ______**

21. **¿Ha tenido dificultades de sueño, en el estado de ánimo u otra de importancia luego de recuperado del Coronavirus?**
Si___ No___
Cuál(es):
____

CARACTERISTICAS DE LA HABITACIÓN PARA DORMIR

22. **Por favor califique entre 1 y 10 el nivel de exposición a la luz de su habitación, durante el tiempo en que usted duerme** (siendo 1 nada exposición y 10 muy expuesto): ______

23. **¿Su habitación para dormir queda cerca de una vía con alto flujo vehicular?** Sí ___ No___

24. **¿La habitación donde duerme está ubicada frente a una zona verde?** Sí___ No___

25. **PREGUNTAS RELACIONADAS CON LA FUNCIONALIDAD FAMILIAR (Apgar Familiar)**

A continuación, lea muy bien cada frase y marque la opción que mejor le represente:

Item	Nunca	Casi nunca	Algunas veces	Casi siempre	Siempre
Me siento satisfecha(o) con el apoyo o la ayuda que recibo de mi familia cuando tengo algún problema y/o necesidad.					
Me siento satisfecha(o) con la forma en que mi familia					

Item	Nunca	Casi nunca	Algunas veces	Casi siempre	Siempre
habla de las cosas y comparte los problemas conmigo.					
Me siento satisfecha(o) con la forma como mi familia acepta y apoya mis deseos de emprender nuevas actividades					
Me siento satisfecha(o) con la forma como mi familia expresa afecto y responde a mis emociones como rabia, tristeza o amor.					
Me siento satisfecha(o) con la manera como compartimos en mi familia: el tiempo para estar juntos, los espacios en la casa y el dinero.					

26. PREGUNTAS RELACIONADAS CON AMENAZAS DE SEGURIDAD PERCIBIDAS EN SU BARRIO

¿Usted se siente inseguro(a) en el Barrio?				
Nunca	Casi nunca	Algunas veces	Casi siempre	Siempre
Si en algun momento se ha sentido inseguro/a en el barrio, señale tres eventos o situaciones graves que podrían ocurrir				
1.<br>2.<br>3.				

Cuestionario sobre Calidad de vida

Instrucciones

A continuación, seleccione un ítem o afirmación por cada pregunta. Por favor escoja la opción que mejor representa HOY su estado de salud o lo que usted siente:

Item	Escala Likert de 1 a 5 (Puntuación)				
1. ¿Cómo calificaría su **calidad de vida**?	Muy mala	Mala	Ni buena ni mala	Buena	Muy buena
2. ¿Qué tan satisfecho está con su **salud**?	Muy insatisfecho	Insatisfecho	Ni satisfecho ni insatisfecho	Satisfecho	Muy satisfecho
3. ¿Qué tan satisfecho está con su capacidad para realizar sus **actividades de la vida diaria**?	Muy insatisfecho	Insatisfecho	Ni satisfecho ni insatisfecho	Satisfecho	Muy satisfecho
4. ¿Qué tan satisfecho está con **usted mismo**?	Muy insatisfecho	Insatisfecho	Ni satisfecho ni insatisfecho	Satisfecho	Muy satisfecho
5. ¿Qué tan satisfecho está con sus **relaciones interperpersonales**: amigos, familiares, conocidos, colegas?	Muy insatisfecho	Insatisfecho	Ni satisfecho ni insatisfecho	Satisfecho	Muy satisfecho
6. ¿Qué tan satisfecho está con las **condiciones de la vivienda**?	Muy insatisfecho	Insatisfecho	Ni satisfecho ni insatisfecho	Satisfecho	Muy satisfecho
7. ¿Se siente con **energía suficiente para sus actividades diarias**?	Para nada	Muy poca	Más o menos	Bastante	Mucha
8. ¿Cuenta con **dinero suficiente** para satisfacer sus necesidades diarias?	Para nada	Muy poco	Más o menos	Bastante	Mucho

Cuestionario sobre calidad del sueño

INTRUCCIONES: las siguientes preguntas se refieren a su forma habitual de dormir únicamente **durante el ÚLTIMO MES**, en promedio. Sus respuestas intentarán ajustarse de la manera más exacta a lo ocurrido durante la mayoría de los días y noches del último mes. Por favor, intente responder a todas las preguntas.

Durante el último mes:

1. ¿A qué hora se acostó normalmente por la noche? **Escriba la hora habitual en que se acuesta: /_____/_____/**

2. ¿Cuánto tiempo se demoró en quedarse dormido en promedio? **Escriba el tiempo en minutos: /___/___/___/**

3. ¿A qué hora se levantó habitualmente por la mañana? **Escriba la hora habitual de levantarse: /_____/_____/**

4. ¿Cuántas horas durmió cada noche? (El tiempo, en este caso, podría ser diferente al que usted permanece en la cama -si lo hace-)
Escriba las horas que crea que durmió: /____/____/

5. Durante el mes pasado, ¿cuántas veces ha tenido usted problemas para dormir a causa de...?

Item	Ninguna vez en el último mes	Menos de una vez a la semana	Una o dos veces a la semana	Tres o más veces a la semana
a. No poder quedarse dormido en la primera media hora.				
b. Despertarse durante la noche o de madrugada.				
c. Tener que levantarse para ir al baño.				
d. No poder respirar bien.				
e. Toser o roncar ruidosamente.				
f. Sentir frío.				
g. Sentir calor.				
h. Tener 'malos sueños' o pesadillas.				
i. Tener dolores.				
j. Otras razones (por favor, descríbalas):				

6. Durante el último mes, ¿cuántas veces ha tomado medicinas (recetadas por el médico o por su cuenta) **para dormir?**
(0) Ninguna vez en el último mes.
(1) Menos de una vez a la semana.
(2) Una o dos veces a la semana.
(3) Tres o más veces a la semana.

7. Durante el último mes, ¿ha tenido problemas para permanecer despierto mientras conducía, comía, trabajaba, estudiaba o desarrollaba alguna otra actividad social?
(0) Nada problemático.
(1) Sólo ligeramente problemático.
(2) Moderadamente problemático.
(3) Muy problemático.

8. Durante el último mes, ¿el 'tener ánimos', qué tanto problema le ha traído a usted para realizar actividades como conducir, comer, trabajar, estudiar o alguna actividad social?
(0) Nada problemático.
(1) Sólo ligeramente problemático.
(2) Moderadamente problemático.
(3) Muy problemático.

9. Durante el último mes, ¿cómo calificaría en conjunto la calidad de su sueño?

(0) Muy buena.
(1) Bastante buena.
(2) Bastante mala.
(3) Muy mala.

10. ¿Tiene usted pareja o compañero/a de habitación?

(0) No tengo pareja ni compañero/a de habitación.
(1) Sí tengo, pero duerme en otra habitación.
(2) Sí tengo, pero duerme en la misma habitación y distinta cama.
(3) Sí tengo y duerme en la misma cama.

Si no tiene pareja o compañero de habitación, no contestar las siguientes preguntas:

Si usted tiene pareja o compañero/a de habitación, pregúntele si usted durante el último mes ha tenido…	**Ninguna vez en el último mes**	**Menos de una vez a la semana**	**Una o dos veces a la semana**	**Tres o más veces a la semana**
a. Ronquidos ruidosos.				
b. Grandes pausas entre respiraciones, mientras duerme.				
c. Sacudidas o espasmos de piernas mientras duerme.				
d. Episodios de desorientación o confusión mientras duerme.				
e. Otros inconvenientes mientras usted duerme; por favor, descríbalos:				

Escala de Zung Parte I

A continuación, lea muy bien cada frase y marque con una **X** (equis) la columna que mejor represente la forma como usted se ha sentido durante la **última semana**.

**Item**	**Nunca o muy pocas veces**	**Algunas veces**	**Muchas veces**	**Siempre o casi siempre**
**1.** Me siento más nervioso de lo normal.				
**2.** Siento miedo sin razón alguna.				
**3.** Me inquieto o atemorizo fácilmente.				
**4.** Siento como que me desintegro y rompo en pedazos.				
**5.** Siento que todo marcha bien y que nada malo puede ocurrir.				
**6.** Mis brazos y piernas se debilitan y tiemblan.				
**7.** Me molestan dolores de cabeza, cuello y espalda.				
8. Me siento débil y me canso fácilmente.				
**9.** Me siento relajado y puedo quedarme tranquilamente sentado con facilidad.				
**10.** Siento que mi corazón late apresuradamente.				
**11.** Tengo sensación de vértigo.				
**12.** Tengo sensación de desmayo o siento como si me fuera a desmayar.				
**13.** Puedo respirar fácilmente.				
**14.** Tengo sensación de adormecimiento y hormigueo en los dedos de las manos y de los pies.				
**15.** Me siento con dolor de estómago o con indigestión.				
**16.** Tengo que orinar con frecuencia.				
**17.** Mis manos permanecen secas y calientes.				
**18.** Mi cara se calienta y siento que me sofoco.				
**19.** Me duermo fácilmente y reposo muy bien durante el sueño.				
**20.** Tengo pesadillas.				

Escala de Zung Parte II

A continuación, lea muy bien cada frase y marque con una **X** (equis) la columna que mejor represente la forma como usted se ha sentido durante las **últimas dos semanas**.

Item	Nunca o muy pocas veces	Algunas veces	Muchas veces	Siempre o casi siempre
1. Me siento triste o deprimido/a				
2. Por las mañanas me siento peor que por las tardes				
3. Frecuentemente tengo ganas de llorar y a veces lloro				
4. Me cuesta mucho dormir o duermo mal por la noche				

5. Ahora tengo menos apetito que antes				
6. Me siento menos atraído/a por el sexo opuesto				
7. Creo que estoy adelgazando				
8. Estoy estreñido/a (constipado/a).				
9. Tengo palpitaciones.				
10. Me canso por cualquier cosa				
11. Mi cabeza no está tan despejada como antes				

Cuestionario sobre percepción del ruido

Por último y por favor a continuación, conteste las siguientes preguntas relacionadas con la percepción que tiene usted del ruido en su vivienda o alrededor de ella:

1. ¿Qué tipos de ruido están presentes en su sector o barrio?

Tráfico Aéreo SI____ NO____
Tráfico Rodado (carros u otros) SI____ NO____
Tráfico Ferroviario SI____ NO____
Industrias SI____ NO____
Actividades de construcción SI____ NO____
Comercio SI____ NO____
Ocio (Bares, Discotecas, fiestas clandestinas) SI____ NO____
Otro (¿cuál?) :____

2. ¿Qué tipo de ruido es más molesto para usted? (seleccionar solo una)

Tráfico Aéreo _____
Tráfico Rodado (carros u otros) _____
Tráfico Ferroviario _____
Industrias_____
Actividades de construcción_____
Comercio_____
Ocio (Bares, Discotecas, fiestas clandestinas) ____
Ninguno ____
Otro (Descrito en la pregunta anterior) :____________________

3. ¿En qué hora u horas del día el ruido se hace más notorio?

A continuación, seleccione una franja(s) horaria por cada día si contestó afirmativamente a la anterior pregunta.

	00:00 - 06:00	06:00 - 12:00	12:00- 18:00	18:00 - 24:00	NA
Lunes					
Martes					
Miércoles					
Jueves					
Viernes					
Sábados					
Domingos					

4. Tomando en consideración el ÚLTIMO MES, indique a usted cuánto le molesta o perturba el ruido (según el ruido más molesto descrito anteriormente) cuando se encuentra en su casa:

Absolutamente nada	Ligeramente	Medianamente	Mucho	Extremadamente

5. **A continuación, se da una escala de opinión de cero a diez para que usted pueda expresar en cuánto le molesta o perturba el ruido (según el ruido más molesto descrito anteriormente) cuando se encuentra en su casa**. Por ejemplo, si usted está "absolutamente nada" molesto por el ruido deberá escoger el cero, y si usted está "extremadamente" molesto debería escoger el diez. Tomando en consideración el ÚLTIMO MES, indique qué número desde el cero al diez expresa mejor que tan molesto o perturbado ha estado usted por dicho ruido cuando se encuentra en su casa:

0	1	2	3	4	5	6	7	8	9	10

6. **¿Su SUEÑO ha sido alterado por el ruido en el último mes?**
Si _____ No _____

7. **Si su respuesta a la pregunta anterior es SI, ¿cuál es la fuente de ese ruido?**
Tráfico Aéreo _____
Tráfico Rodado (carros u otros) _____
Tráfico Ferroviario _____
Industrias_____
Actividades de construcción_____
Comercio_____
Ocio (Bares, Discotecas, fiestas clandestinas) ____
Otro (¿cuál?) :____________________

8. **Tomando en consideración el ÚLTIMO MES, indique a usted cuánto le afecta el ruido en su CALIDAD DE VIDA:**

Absolutamente nada	Ligeramente	Medianamente	Mucho	Extremadamente

9. **A continuación, se da una escala de opinión de cero a diez para que usted pueda expresar cuánto le afecta el ruido en su CALIDAD DE VIDA**. Por ejemplo, si usted está "absolutamente nada" afectado en su calidad de vida por el ruido en su casa deberá escoger el cero, y si usted está "extremadamente" afectado en su calidad de vida por el ruido en su casa debería escoger el diez. Tomando en consideración el ÚLTIMO MES, indique qué número desde el cero al diez expresa mejor que tan afectada ha estado su calidad de vida por el ruido en su casa

0	1	2	3	4	5	6	7	8	9	10

10. ¿Cuál es el ruido que más le incomoda dentro de su casa?
Lavadora ____
Televisor prendido____
Licuadora____
Ninguno ____
Otro (¿cuál?) :____________________

11. ¿El ruido al que está expuesto proviene mayormente de las fuentes externas a su casa?
Por favor marque con una x

Nunca	Casi nunca	Algunas veces	Casi siempre	Siempre

12. ¿Qué tanto le afecta el ruido que causan los aparatos electrónicos con los que interactúa diariamente dentro de su casa?
Por favor marque con una x

Nunca	Casi nunca	Algunas veces	Casi siempre	Siempre

13. ¿Cómo cambió su percepción del ruido exterior durante el confinamiento generado por el covid-19?

Aumentó _____ Disminuyó _____

0%	20%	40%	60%	80%	100%

14. ¿Cómo ha cambiado su percepción del ruido exterior con la reactivación de la economía y la implementación de la "nueva normalidad"?

Aumentó _____ Disminuyó _____

0%	20%	40%	60%	80%	100%

15. ¿Categoría de exposición a ruido según mapa histórico (pregunta según dato referido por el encuestador)?
Baja___ Alta ___

Agradecemos su participación voluntaria en este estudio
